# Genotype‐environment associations support a mosaic hybrid zone between two tidal marsh birds

**DOI:** 10.1002/ece3.1864

**Published:** 2015-12-29

**Authors:** Jennifer Walsh, Rebecca J. Rowe, Brian J. Olsen, W. Gregory Shriver, Adrienne I. Kovach

**Affiliations:** ^1^ Department of Natural Resources and the EnvironmentUniversity of New HampshireDurhamNew Hampshire; ^2^ School of Biology and EcologyUniversity of MaineOronoMaine; ^3^ Department of Entomology and Wildlife EcologyUniversity of DelawareNewarkDelaware

**Keywords:** *Ammodramus caudacutus*, *Ammodramus nelsoni*, ecological niche models, genotype‐habitat associations, hybridization, mosaic hybrid zone, Nelson's sparrow, Saltmarsh sparrow

## Abstract

Local environmental features can shape hybrid zone dynamics when hybrids are bounded by ecotones or when patchily distributed habitat types lead to a corresponding mosaic of genotypes. We investigated the role of marsh‐level characteristics in shaping a hybrid zone between two recently diverged avian taxa – Saltmarsh (*Ammodramus caudacutus*) and Nelson's (*A. nelsoni*) sparrows. These species occupy different niches where allopatric, with *caudacutus* restricted to coastal marshes and *nelsoni* found in a broader array of wetland and grassland habitats and co‐occur in tidal marshes in sympatry. We determined the influence of habitat types on the distribution of pure and hybrid sparrows and assessed the degree of overlap in the ecological niche of each taxon. To do this, we sampled and genotyped 305 sparrows from 34 marshes across the hybrid zone and from adjacent regions. We used linear regression to test for associations between marsh characteristics and the distribution of pure and admixed sparrows. We found a positive correlation between genotype and environmental variables with a patchy distribution of genotypes and habitats across the hybrid zone. Ecological niche models suggest that the hybrid niche was more similar to that of *A. nelsoni* and habitat suitability was influenced strongly by distance from coastline. Our results support a mosaic model of hybrid zone maintenance, suggesting a role for local environmental features in shaping the distribution and frequency of pure species and hybrids across space.

## Introduction

Hybrid zones are considered windows onto the evolutionary process (Harrison 1990), providing unique environments for investigating the mechanisms driving reproductive isolation and the role of these processes in generating and preserving biodiversity. Understanding how species are maintained in the face of ongoing hybridization and introgression can elucidate processes fundamental to speciation. Temporally stable hybrid zones are maintained by a balance between dispersal of parental taxa into a zone and selection against hybrids (Haldane 1948; Barton and Hewitt 1985). The selective forces responsible for shaping zone dynamics within a stable hybrid zone, however, can vary. Selection against hybrids can take many forms, but is often broadly categorized as either environment‐independent (endogenous) or environment‐dependent (exogenous). Often these forces are not mutually exclusive, and a range of factors, including habitat affinity, behavior, and fitness can shape hybrid zone dynamics within a natural system. Identifying the relative influence of these selective forces can provide new insights into the role and function of isolating mechanisms and their relative predominance across taxa and systems (e.g., Bronson et al. 2003; Hamilton et al. 2013; Tarroso et al. 2014).

While the role of environment‐independent selection (i.e., genetic incompatibilities) is unarguably a critical factor in shaping dynamics across natural hybrid zones, a growing body of research suggests an important and potentially overlooked role for the environment in the regulation of both plant and animal hybrid zones (Carson et al. 2012; Culumber et al. 2012; De La Torre et al. 2014; Tarroso et al. 2014). Hybrid zones often occur along ecological gradients, as transitional habitats may facilitate contact between species occupying different ecological niches (Culumber et al. 2012). In cases of environment‐dependent selection, the spatial distribution of individuals within a hybrid zone should correlate strongly with their genotypes (Arnold 1997; Johnston et al. 2001). These genotype‐habitat associations may arise from habitat preferences or from differential fitness in adjacent habitat types (Arnold 1997). The Bounded Hybrid Superiority model (Moore 1977) predicts that hybrid distribution will be spatially bounded within an ecologically intermediate area, where hybrid genotypes are more fit relative to parental forms. Conversely, the Mosaic Hybrid Zone model (Harrison and Rand 1989; Rand and Harrison 1989) predicts that the spatial distribution of pure and hybrid genotypes may be highly variable as a result of adaptation of parental forms to two different and patchily distributed environments. In both models, local environmental features may be particularly influential in shaping hybrid zones that have formed between recently diverged taxa, for which postzygotic barriers, including hybrid inviability, may be slower to evolve (e.g., avian systems; Fitzpatrick 2004).

In this study, we investigated the relationship between habitat and genotype across a naturally occurring hybrid zone between Saltmarsh (*Ammodramus caudacutus*) and Nelson's sparrows (*A. nelsoni*). *A. caudacutus* is a habitat specialist, exhibiting a pre‐Pleistocene association with tidal salt marshes (Greenlaw and Rising 1994; Chan et al. 2006). In contrast, *A. nelsoni* exhibits a broader ecological niche, breeding in grassland and brackish marshes in addition to tidal marshes (Greenlaw 1993; Nocera et al. 2007; Shriver et al. 2011). These recently diverged (~600,000 years; Rising and Avise 1993; Klicka et al. 2014) sister species have come into secondary contact in the northeastern United States likely following the last glacial recession (Rising and Avise 1993). In the USA and Maritime Canada, *A. caudacutus* and *A. nelsoni* are restricted to a ribbon of tidal marsh habitat along the Atlantic seaboard with a subspecies of *caudacutus* (*A.c. caudacutus*) inhabiting coastal salt marshes from southern Maine to New Jersey and a subspecies of *nelsoni* (*A.n. subvirgatus*) inhabiting brackish and tidal marshes from the Canadian Maritimes to northern Massachusetts (Greenlaw and Rising 1994; Shriver et al. 2011). The two subspecies (from here on referred to as *caudacutus* and *nelsoni*) overlap and hybridize along a 210 km stretch of the New England coast between the Weskeag River estuary in South Thomaston, Maine and Plum Island in Newburyport, Massachusetts (Hodgman et al. 2002; Shiver et al. 2005, Walsh et al. 2011). While admixture is extensive throughout the hybrid zone (Walsh et al. 2015), the genetic structure across sympatric populations is patchy (Walsh et al. in review), suggesting a potential role for habitat associations. Here, we explore the role of local environmental features in shaping the frequency and distribution of pure and hybrid individuals across the *caudacutus‐nelsoni* hybrid zone. The spatial distribution of tidal marshes along the coastline coupled with their characteristic adaptive gradient provides an ideal system for investigating patterns of environment‐dependent selection. Furthermore, understanding the role of habitat in shaping interspecific interactions within this system has broader management implications, as both species are a high conservation priority in the Northeast due to their limited range and vulnerability to habitat loss (USDI 2008).

The *caudacutus‐nelsoni* hybrid zone corresponds geographically to a habitat discontinuity along the coastline, with a transition from smaller, isolated, and more brackish fringe marshes in the north (pure *nelsoni* habitat) to more expansive, continuous stretches of tidally influenced marshes in the south (pure *caudacutus* habitat; Greenlaw 1993). Variation in habitat affinity between the two species suggests a role for local environmental features as a potentially important isolating mechanism. Abrupt environmental gradients across the marine‐terrestrial ecotone within each marsh present adaptive challenges to terrestrial vertebrates (*e.g*., tidal inundation and osmoregulatory demands; Goldstein 2006, Bayard and Elphick 2011), and provide unique opportunities to investigate evolutionary processes (Greenberg 2006). While there is a linear, latitudinal transition between the brackish upriver (North – *nelsoni*) and primarily coastal (South – *caudacutus*) marsh types, the intervening habitat found within the hybrid zone is characterized by a mix of marsh types. This complex spatial structuring of tidal marsh habitat within the hybrid zone may result in a corresponding mosaic of genotypes.

Here, we investigated the role of habitat as a potential mechanism responsible for shaping dynamics across the *caudacutus‐nelsoni* hybrid zone by evaluating the role of local habitat features in shaping the distribution of pure and admixed individuals. We hypothesized that the environmental gradients characteristic of salt marsh ecosystems would influence the distribution of these two differentially adapted taxa and the level and direction of introgression. If environment‐dependent selection plays a role in maintaining the hybrid zone, we expect that the spatial distribution of individuals within the hybrid zone should correlate strongly with their genotypes (Arnold 1997). To test this hypothesis, we employed a combination of genetic and geospatial techniques to characterize both genotypic and environmental variation across the full extent of the hybrid zone. Specifically, we tested these predictions by (1) evaluating the distribution of pure and admixed individuals in relation to environmental characteristics, and (2) assessing differences in the ecological niche space of pure species and hybrids.

## Methods

### Sample collection

**Figure 1:**
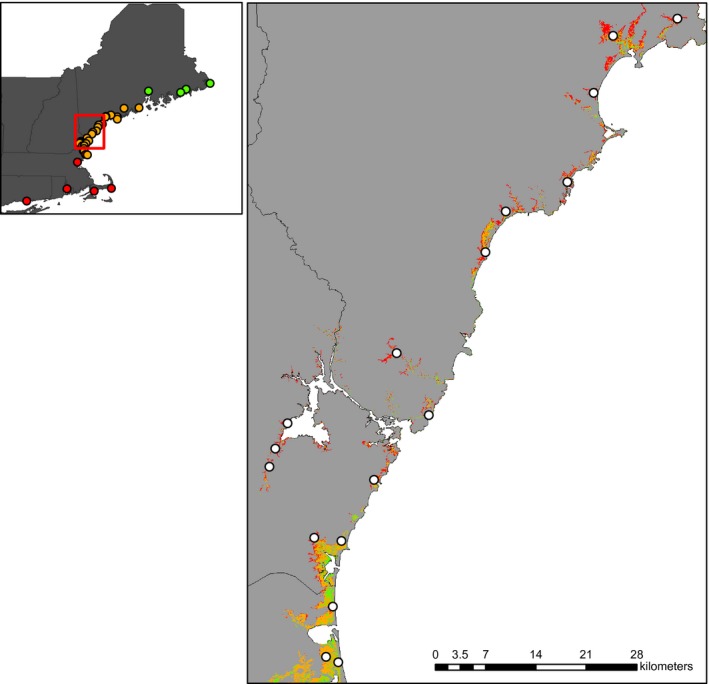
Map of *nelsoni* and *caudacutus* sampling locations. Transect sampling locations are shown on the map inset: allopatric *nelsoni* points are in green, sympatric locations are orange, and allopatric *caudacutus* points are red. The larger map shows an example of the marsh patch layer with sampling locations indicated by white circles. Colored areas of map indicate vegetation type (high marsh in red, mixed marsh in orange, and low marsh in green).

To capture the extent of genetic variation across the hybrid zone, we sampled 305 sparrows from 34 marshes along a linear transect from Lubec, Maine to Madison, Connecticut (Fig. 1; Table 1) during the 2012 and 2013 breeding seasons (June – August). Tidal marshes are unique in that they are discrete habitat patches that occur in a narrow ribbon along the coastline. This spatial arrangement provides a relatively simple experimental design whereby a linear transect captures the full extent of variation in pure and admixed populations within and surrounding the hybrid zone. We sampled marshes across the hybrid zone approximately every 10 km (*n *=* *23) and included four allopatric *nelsoni* marshes and seven allopatric *caudacutus* marshes. We deployed three to six 12‐m mist nets with 30 mm mesh to capture a target sample of 10 birds from each site. We collected blood samples (10–20 *μ*L) from the brachial vein and transferred samples to Nobuto blood filter strips (Sterlitech, Kent, Washington) where they were stored at room temperature for later genetic analysis.

**Table 1:** Sampling locations for *nelsoni* and *caudacutus* individuals. Table includes a site code for each marsh, the marsh name, coordinates, number of individuals sampled, and whether the marsh was considered allopatric *nelsoni*, sympatric, or allopatric *caudacutus*. Descriptive environmental features are included for each marsh (patch size in hectares, proximity index, and distance of marsh to the nearest ocean shoreline in meters)

Site code	Locality	Latitude	Longitude	*N*	Population	Marsh size (ha)	Proximity index	Distance to shoreline (meters)
1	Lubec, ME	44.822	−66.991	9	Allopatric *nelsoni*	15.4	0.000	9852.00
2	Columbia Falls, ME	44.644	−67.719	10	Allopatric *nelsoni*	123.2	0.200	11,246.00
3	Narraguagus River – Millbridge, ME	44.551	−68.891	9	Allopatric *nelsoni*	66.8	0.067	14,981.00
4	Mendell Marsh – Penobscot, ME	44.591	−68.859	9	Allopatric *nelsoni*	118.2	0.002	5385.00
5	Weskeag Marsh – South Thomaston, ME	44.077	−69.142	9	Sympatric	128.3	0.006	5795.10
6	Sheepscot River – Newcastle, ME	44.065	−69.597	7	Sympatric	99.9	0.192	16,196.61
7	Morse Cove – Arrowsic, ME	43.816	−69.795	5	Sympatric	71.02	0.133	6696.00
8	Popham Beach – Phippsburg, ME	43.739	−69.806	15	Sympatric	143	0.294	793.97
9	Maquoit Bay – Brunswick, ME	43.867	−69.988	10	Sympatric	27.9	0.051	107.64
10	Cousins River – Yarmouth, ME	43.811	−70.156	5	Sympatric	65.2	0.023	5156.00
11	Spurwink River – Cape Elizabeth, ME	43.588	−70.246	16	Sympatric	261.2	0.576	3046.00
12	Scarborough Marsh – Scarborough, ME	43.575	−70.372	14	Sympatric	959	0.426	3216.41
13	Saco River – Saco, ME	43.492	−70.391	7	Sympatric	61.7	0.078	516.60
14	Marshall Point – Arundel, ME	43.381	−70.433	6	Sympatric	160.8	0.067	701.53
15	Little River – Wells, ME	43.344	−70.538	4	Sympatric	86.2	0.498	735.14
16	Eldridge Marsh – Wells, ME	43.292	−70.572	9	Sympatric	414	0.733	195.09
17	York River – York, ME	43.161	−70.732	2	Sympatric	135	0.018	7496.66
18	Seapoint – Kittery Point, ME	43.087	−70.664	9	Sympatric	21.3	0.402	108.85
19	Lubberland Creek – Newmarket, NH	43.073	−70.903	10	Sympatric	22.4	0.150	15,246.00
20	Chapman's Landing – Stratham, NH	43.041	−70.924	10	Sympatric	86.9	0.112	14,352.73
21	Squamscott River – Exeter, NH	43.017	−70.935	6	Sympatric	75.24	0.080	15,440.09
22	Awcomin Marsh – Rye, NH	43.006	−70.752	7	Sympatric	78.9	0.591	748.18
23	Drakeside Marsh – Hampton, NH	42.931	−70.852	7	Sympatric	1775.8^1^	8.329^1^	4709.38
24	Hampton Beach – Hampton, NH	42.926	−70.806	9	Sympatric	1775.8^1^	8.329^1^	903.08
25	Salisbury Marsh – Salisbury, MA	42.844	−70.822	10	Sympatric	1775.8^1^	8.329^1^	352.66
26	Pine Island – Newburyport, MA	42.775	−70.827	13	Sympatric	781^2^	3.061^2^	2129.04
27	Plum Island – Newburyport, MA	42.774	−70.809	9	Sympatric	781^2^	3.061^2^	595.36
28	Castle Hill – Ipswich, MA	42.679	−70.773	7	Allopatric *caudacutus*	746.4	2.407	873.49
29	Farm Creek Marshes – Gloucester, MA	42.658	−70.708	10	Allopatric *caudacutus*	75.9	0.575	403.37
30	Revere, MA	42.436	−71.011	5	Allopatric *caudacutus*	292.7	0.021	2876.10
31	Monomoy Island – Chatham, MA	41.603	−69.987	11	Allopatric *caudacutus*	36.3	0.000	115.54
32	Waquoit Bay – Mashpee, MA	41.555	−70.506	2	Allopatric *caudacutus*	28.2	0.164	400.00
33	Prudence Island – Jamestown, RI	41.647	−71.343	9	Allopatric *caudacutus*	31.9	0.330	527.70
34	Hammonasset Beach – Madison, CT	41.263	−72.551	10	Allopatric *caudacutus*	347.21	0.213	324.07

a Same marsh complex.

### Quantifying environmental variation

Sampling efforts covered a diversity of marsh patches to evaluate the relationship among the distribution of *nelsoni*,* caudacutus*, their hybrids, and environmental variables. Marshes varied in size, tidal regimes, and connectivity to neighboring patches (Table 1). We collected all samples within saline or brackish marshes (euhaline to oligohaline); however, the location of those marshes varied and included coastal salt marshes adjacent to the ocean, tidal marshes in bay systems, and smaller fringe marshes farther up river (Fig. 1). We measured a suite of environmental variables to describe the differences between pure *nelsoni* and pure *caudacutus* habitat types including marsh size, isolation, and tidal influence (Table 2). We tested for the correlation of site‐specific genotypes with seven local variables (size, patch isolation, proportion of high marsh, proportion of low marsh, NDVI, distance to upland edge, and distance to shoreline) to determine genotype‐habitat associations; and we used four variables (shoreline distance, marsh isolation, NDVI, and vegetation type) to assess niche similarities with ecological niche models (Table 2).

**Table 2:** Habitat features quantified for each sampling location and included in this study, including marsh size, proximity to neighboring marshes, Normalized Difference Vegetation Index (NDVI), proportion of low (predominantly *Spartina alterniflora*), and high marsh (*Spartina patens* and *Juncus gerardii*; vegetation is expressed both separately for genotype‐habitat associations or compiled as a vegetation map for the ecological niche models), and distance to shoreline and upland edge. Table includes the habitat feature measured, rational for each measurement (see text), whether the habitat variable was used for genotype‐habitat associations (GHA) or ecological niche models (ENM), the scale at which the variable was collected, and the mean and range of values for each variable

Habitat feature	Prediction/rationale	Analysis type	Scale	Mean/range
Marsh size	*nelsoni* found in smaller marshes compared to *caudacuts*	GHA	Marsh complex	335 ha/15–1775 ha
Proximity index/proximity surface	*nelsoni* found in more isolated marshes compared to *caudacuts*	GHA/ENM	Marsh complex/study area	0.985/0 – 8.1
NDVI (max and average)	*nelsoni* found in marshes with a higher average NDVI	GHA/ENM	Point of capture/study area	0.08/0–0.29
Proportion of low marsh (*S. alterniflora*)	*nelsoni* found in marshes with a smaller proportion of low marsh	GHA/ENM	Point of capture	8.57%/0–58%
Proportion of high marsh (*S. patens* and *J. gerardii*)	*nelsoni* found in marshes with a larger proportion of high marsh	GHA/ENM	Point of capture	43.65%/0–100%
Distance to upland	*nelsoni* found in up river fringe marshes closer to upland edge compared to *caudacutus*	GHA	Point of capture	218 m/14–1551 m
Shoreline distance/shoreline surface	*nelsoni* found further from ocean shoreline	GHA/ENM	Marsh complex/study area	4078 m/84.2–16,196 m

For genotype‐habitat associations, we quantified habitat variables at both the marsh complex and a point‐of‐capture scale. For the marsh complex scale, we defined marsh patches as stretches of continuous marsh separated from neighboring marsh by >50 m of upland habitat or >500 m of open water (Benoit and Askins 2002), and measured marsh size, distance to ocean shoreline, and isolation for the entire patch. We measured marsh size using fragstats version 4 (McGarigal et al. 2012). We quantified marsh isolation by calculating a proximity index following the methods of Gustafson and Parker (1994) using three buffer sizes: 1, 5, and 10 km. Briefly, the proximity index is calculated by measuring the shortest linear distance from the focal marsh to the edge of all adjacent marshes within the buffer, dividing the area of each adjacent marsh by its distance from the focal marsh, and summing these values for all marshes within the buffer (values range from 0 to 10, with 0 being completely isolated). We measured distance to shoreline as the minimum distance between the marsh patch and the nearest ocean shoreline (defined using vector layers from USGS National Assessment of Shoreline Change and National Geodetic Survey Coastal Mapping Program).

**Figure 2:**
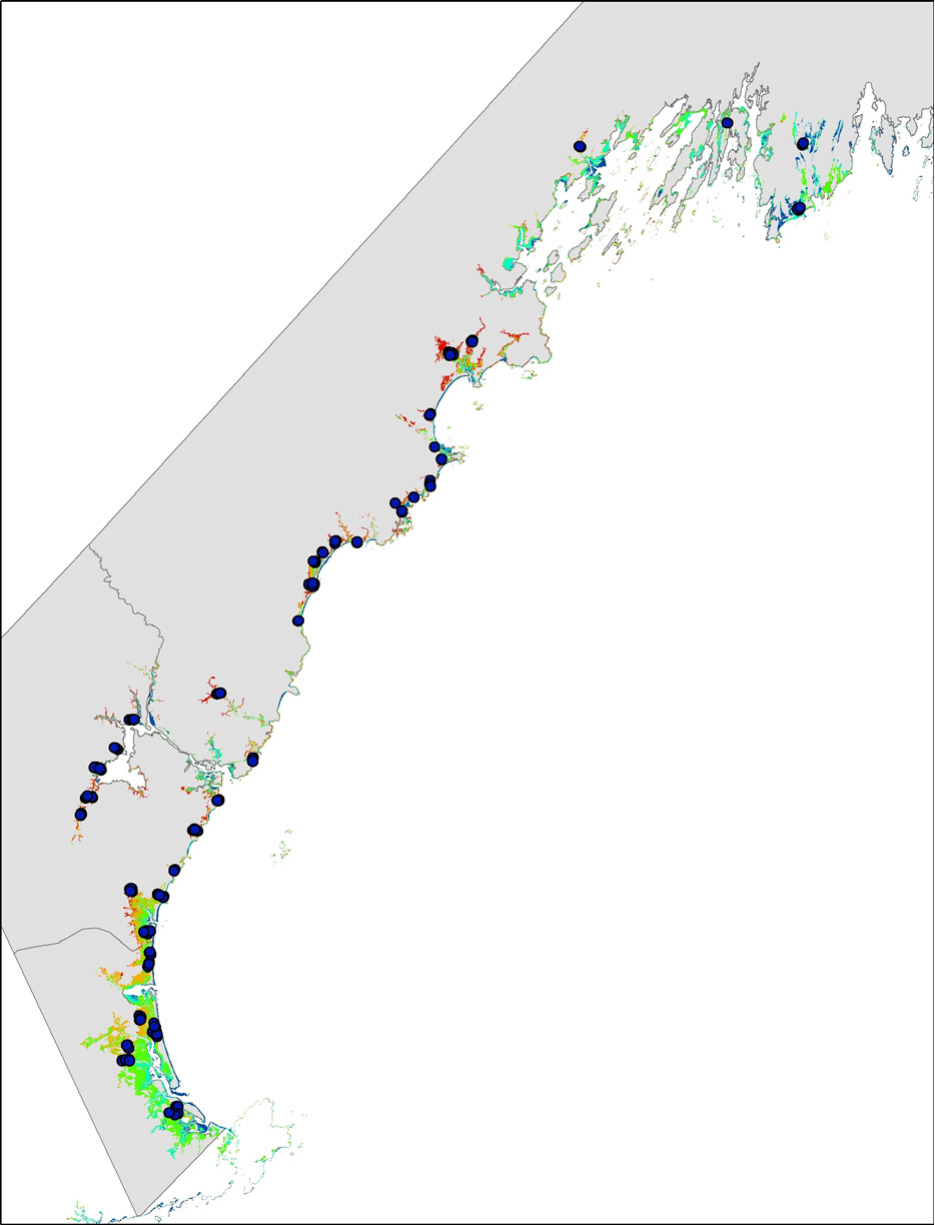
Map showing field evaluation points for assessing the accuracy of the vegetation map. At each point, vegetation composition was visually inspected and recorded as high, low, or mixed marsh. A point was considered high marsh if it contained greater than 70% high marsh or low marsh if it contained greater than 70% low marsh. Areas were considered mixed if they contained relatively equal proportions of high/low marsh vegetetation. Classification of field vegetation points were then compared to vegetation composition predicted by the remote sensed map.

At the point‐of‐capture scale, we collected data on vegetation and distance to upland edge within a 5.25‐ha buffer around the bird capture locations that correspond to the average core home range size of a female Saltmarsh Sparrow (which have the smallest core area of both species, males and females; Shriver et al. 2010). We developed a vegetation map for the study area that reflected three major vegetation zones: high marsh (inundated only during the monthly high tides), low marsh (inundated daily), and mixed marsh in order to quantify local tidal regime at our sampling locations. To do this, we obtained five Landsat ETM satellite images (30‐m spatial resolution), acquired in July–September of 2000–2002, which covered the entire study area; we did not use any images taken at peak high tide (when most of the marsh is inundated with water). We calculated the Normalized Difference Vegetation Index (NDVI), which ranges from −1 to 1, with negative values corresponding to an absence of vegetation (Myneni et al. 1995). We used NDVI to differentiate between inundated areas or low marsh (low NDVI values) and vegetated areas or high marsh (higher NDVI values). Using NDVI values for the study area as input, we ran an Iso Cluster unsupervised classification in AR‐CMAP v10 (ESRI, Redlands, CA, USA) to assign pixels to one of five classes: (1) water, (2) pools, (3) low marsh, (4) mixed marsh, and (5) high marsh. We evaluated the accuracy of the vegetation map by visiting 137 random points within the study area and comparing map classification to field classification (Fig. 2). Overall classification accuracy of the resulting vegetation map was 72% (Table 3) and was therefore suitable for subsequent analyses. We calculated maximum and average NDVI and proportion of high and low marsh from the vegetation map at the point‐of‐capture scale for genotype‐habitat associations. We also used latitude as a covariate in the genotype‐habitat associations.

**Table 3:** Summary of vegetation classification accuracy for the remote sensed map. Table includes total number of points for each category and the number of points correctly classified based on field visits

Vegetation class	Map classification – total # of points	Field classification – # of points correctly classified	% Accuracy
High/mixed marsh	91	63	69%
Low marsh	35	25	71%
Water (open/pools)	11	11	100%
Total	137	99	72%

For ecological niche models, we included four environmental variables over a continuous spatial extent covering the entire study area: distance to shoreline, marsh isolation, NDVI, and vegetation zones, characterizing vegetation as high, mixed, and low marsh (Table 2). We calculated the distance to shoreline by creating a continuous Euclidean distance surface using the same shoreline vector layers (see above) as input in arcmap v10 (ESRI). Similarly, to quantify patch isolation, we created another continuous Euclidean distance surface that represented the distance between suitable habitats using all marsh patches within the study area as input. We also included vegetation maps and raw NDVI values for the entire study area in the niche models.

### Genetic data and admixture analysis

To evaluate the role of local habitat features in shaping the distribution of pure and admixed individuals, we used genotypes from 24 microsatellite loci from a previously published genetic data set (Walsh et al. 2015) to calculate a site‐averaged genotype for correlation with measured environmental features. This final data set included genotypes from 290 individuals (Table 1). To obtain a site‐averaged genotype, we first characterized spatial variation in allele frequencies using principal components analysis (PCA; Patterson et al. 2006) of the multilocus genotypes with the prcomp function in R (R Development Core Team 2014). Eigenvectors for all PCAs (genotype and habitat; see below) were rotated using varimax rotation (Krzanowski 2000). Principal component one (PC1) explained 42% of the variation and reflected the relative contribution of *nelsoni* and *caudacutus* alleles to an individual genotype (negative scores were representative of *nelsoni* genotypes and positive scores representative of *caudacutus* genotypes). Individual PC1 scores were averaged for each sampling location, representing the average allelic composition of a population. All individuals were additionally assigned to one of five genotypic classes (pure, backcrossed, F1/F2) following the approach in Walsh et al. (2015; Appendix S1).

### Genotype‐habitat associations

**Figure 3:**
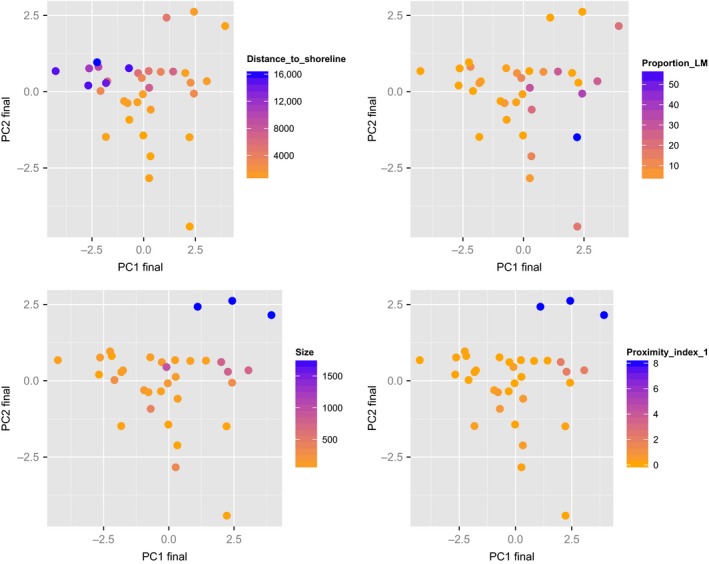
Plot of first and second axis of PCA of all 11 habitat variables. Each plot shows PC1 and PC2 scores for 34 marshes and are color coded to represent each of the four most influential variables: distance to shoreline, proportion of low marsh (LM), marsh size, and proximity index. Cumulatively, these plots indicate that marshes with higher PC1 scores are closer to the shoreline, have a greater proportion of low marsh, are larger, and closer to adjacent marshes.

**Figure 4:**
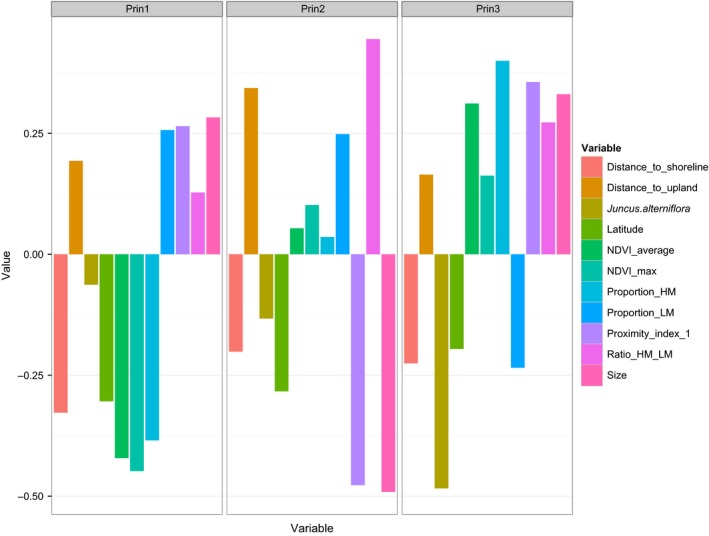
Loadings for principal components (PCs) one through three for 11 environmental variables.

We tested for correlations between habitat variables and genotype to assess whether the distribution of pure and admixed individuals was dependent on environmental features at the point‐of‐capture (vegetation) and marsh complex (size, proximity, distance to shoreline) scale. Based on our predictions for a mosaic hybrid zone, we expected to see a correlation between habitat type and genotype. To test for this, we used a PCA of all habitat variables (marsh complex and point‐of‐capture scale) to identify the features that were most informative in explaining variation in marsh habitats. To capture differences between the marsh complex and point‐of‐capture scale, we also performed a PCA on variables collected at these two scales separately. Because PC scores appear to roughly separate marshes based on tidal regime, vegetation composition, size, and isolation (Figs. 3, 4), we used the distribution of PC1 scores (all habitat variables collected over both spatial scales) to broadly classify marshes as coastal, river, and intermediate. Marshes that were smaller, dryer, farther from the coastline, and more isolated were associated with the negative side of PC1 and represented upriver marshes. Marshes that were larger, wetter, closer to the coastline, and more continuous were associated with the positive side of PC1 and represented coastal marshes. To ensure that there were clear habitat differences among the sites and that latitude was not the main driver of the PCA results, we also ran a PCA excluding latitude. The same proportion of the variation was explained with and without latitude and thus we removed latitude for subsequent analyses. To relate marsh‐level allele frequencies to habitat variation, we used the site‐averaged allele frequency scores from the genotype PCA as a dependent variable in a linear regression. We used the scores from the PC1 axis of the local and marsh complex habitat variables (both separately and combined) as predictor variables. We used the mass package in R for these analyses.

### Ecological niche models

We used ecological niche modeling to assess differences in the niche space of pure *caudacutus, nelsoni,* and hybrids. Based on our predictions for a mosaic hybrid zone, we expected to see variation in niche space for pure and hybrid individuals and a patchy distribution of both upriver and coastal marsh habitat types and genotypes across the hybrid zone. We developed ecological niche models for each parental species and hybrids using a maximum entropy method implemented in the program Maxent v.3.3.2 (Phillips et al. 2006; Phillips and Dudik 2008). We input occurrence data for pure *nelsoni* (*n *=* *94) and pure *caudacutus* (*n *=* *83) from our transect sampling efforts (points used only if genetically pure individuals were identified at a site) and from a range‐wide survey (points used only if outside of the putative hybrid zone; Wiest et al. in review, Appendix S2). Hybrid occurrence points (*n *=* *23) included transect sampling sites where individuals were classified as admixed based on multilocus genotypes (Appendix S2). Because both species are marsh specialists, the four environmental data layers (described above) were clipped to include only marsh habitat in our study area – Maine to Connecticut.

Maxent uses environmental data from known occurrence points to predict the expected distribution of a species and produces a map where each grid cell represents the predicted suitability for each species. The performance of each model is estimated based on the area under the receiver operating curve (AUC); higher AUC values indicate better predictive ability of a model whereas values equal to 0.5 indicate that a model performed no better than random (Phillips and Dudik 2008). We ran Maxent with 10 replicate runs per species and 5000 iterations using the K‐fold cross‐validation method (K = 10; Phillips et al. 2006). We averaged AUC values across the 10 replicates for each species and considered models with a mean AUC ≥ 0.7 to be informative (Swets 1998). We developed a threshold value for suitable versus unsuitable habitat following the methods of Chatfield et al. (2010). We examined the cumulative probabilities associated with each occurrence point and classified any grid cell falling in the lower 5th percentile of this distribution as unsuitable habitat. We used the program ENMTools (Warren et al. 2008, 2010) to quantify the amount of niche overlap between *nelsoni*,* caudacutus*, and hybrids. ENMTools employs two measures for niche overlap, Schoener's *D* and Warren's *I*, both of which range from 0 (no niche overlap) to 1 (complete overlap).

## Results

### Habitat variation

Marsh characteristics differed between pure *nelsoni* and *caudacutus*: on average, marshes dominated by *nelsoni* genotypes (*nelsoni* marshes) were smaller, more isolated, and dryer than marshes dominated by *caudacutus* genotypes (*caudacutus* marshes). Average size and proximity indices were 80 ha (SE ± 25 ha) and 0.01 (±0.01), respectively, for pure *nelsoni* marshes compared to 222 ha (±100 ha) and 0.42 (±0.27) for pure *caudacutus* marshes. The average proportion of low marsh was also lower in the pure *nelsoni* marshes (2.0 ± 2.21%) compared to the pure *caudacutus* marshes (23.0 ± 8.8%). A PCA of all measured habitat variables identified two axes that explained a majority of the environmental variation (34% and 17%; Table 4). PC1 was highly correlated with NDVI, ratio of high marsh, and distance to shoreline – variables indicative of tidal regime. PC2 was highly correlated with marsh size and proximity – variables indicative of patch‐level characteristics (Fig. 4).

**Table 4:** Factor loadings for the top two principal components (PCs) resulting from a Principal Component Analysis (PCA) of habitat variables. Rationale for the habitat variables is outlined in Table 2. Factor loadings describe local habitat variation among marshes sampled for *A. nelsoni* and *A. caudacutus* individuals

Variable	PC1	PC2
Size	0.28	−0.49
Proximity index	0.27	−0.47
Proportion of low marsh	0.25	0.25
Distance to upland	0.19	0.34
Ratio of high to low marsh	0.13	0.44
*juncus* to *alterniflora*	−0.06	−0.13
Latitude	−0.31	−0.28
Distance to shoreline	−0.33	−0.21
Proportion of high marsh	−0.38	0.03
NDVI average	−0.42	0.05
NDVI max	−0.44	0.11
Eigenvalue	1.98	1.38
% Variance	34	17

### Genotype‐habitat associations and ecological niche models

**Figure 5:**
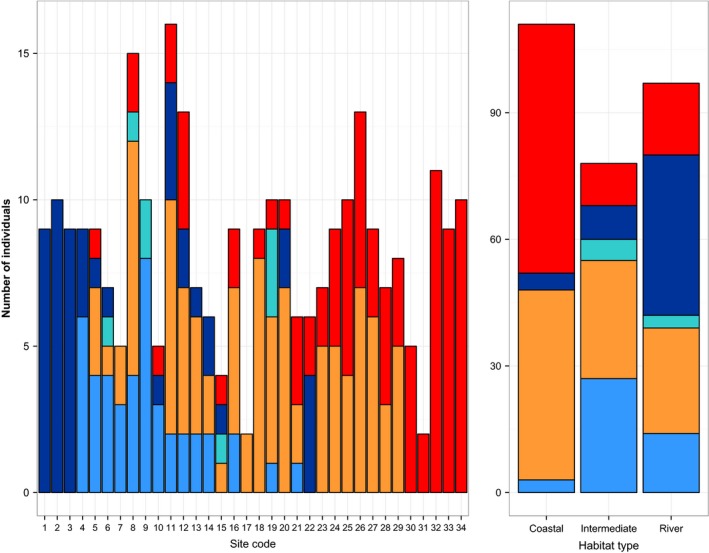
Distribution of genotypic classes by sampling location (left) and by habitat type (right). Left panel shows the distribution of genotypic classes from Lubec, Maine (site code 1) to Madison, Connecticut (site code 34) and right panel shows the distribution of genotypic classes in coastal, intermediate, and river marshes (based on distribution of PC scores for habitat variables; see text). Genotypic classes are color coded as follows: pure *caudacutus* (red), backcrossed *caudacutus* (orange), F1/F2 (teal), backcrossed *nelsoni* (light blue), and pure *nelsoni* (dark blue).

A comparison of pure and hybrid distribution across marsh types revealed that F1/F2 hybrids were found only in upriver or intermediate marshes (those with intermediate PC1 scores; see Methods) as opposed to coastal marshes (Fig. 5). We found that upriver and intermediate marshes were also characterized by higher diversity of genotypic classes, with a more even distribution of pure and backcrossed individuals. Coastal marshes were characterized by high proportions of pure and backcrossed *caudacutus* (Fig. 5).

**Figure 6:**
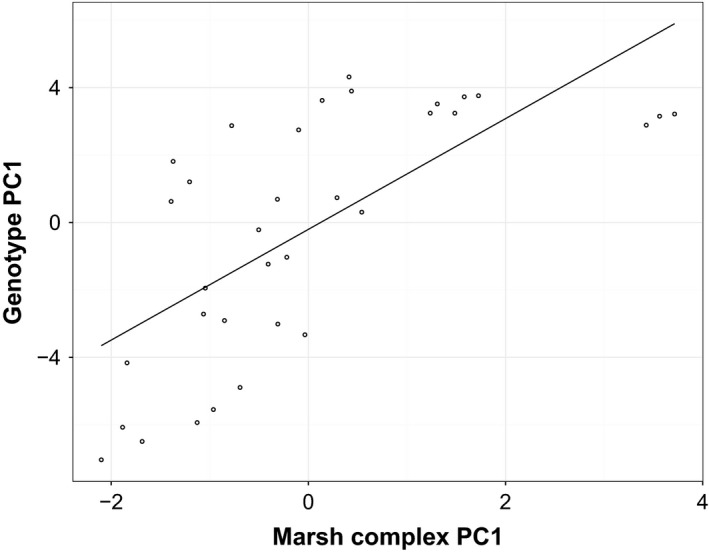
Correlation between habitat PC1 scores at the marsh complex scale (distance to shoreline, distance to upland, size, proximity) and genotype PC1 scores. Negative scores are more representative of *nelsoni* alleles (genotype PC) and fringe marshes (habitat PC) and positive scores are representative of *caudacutus* alleles (genotype PC) and coastal marshes (habitat PC).

Habitat variables across the two spatial scales (point‐of‐capture and marsh complex) explained the distribution of *caudacutus* and *nelsoni* alleles across the study area (*R* ^2^ = 0.35, *P* < 0.001). Marsh complex characteristics (size, proximity, distance to shoreline, and distance to upland) explained more of the variation in allelic distribution (*R* ^2^ = 0.45, *P* < 0.001; Fig. 6) than point‐of‐capture (local vegetation) characteristics (*R* ^2^ = 0.10, *P* = 0.03). Parental species occurred with greatest frequency at the extremes along PC axis one, which described the transition from upriver to coastal marshes (Fig. 3). When allopatric populations were removed, both the full suite of environmental variables (*R* ^2^ = 0.13, *P* = 0.04) and the marsh complex characteristics (*R* ^2^ = 0.34, *P* = 0.001) explained the distribution of *caudacutus* and *nelsoni* alleles across the sympatric populations.

**Figure 7:**
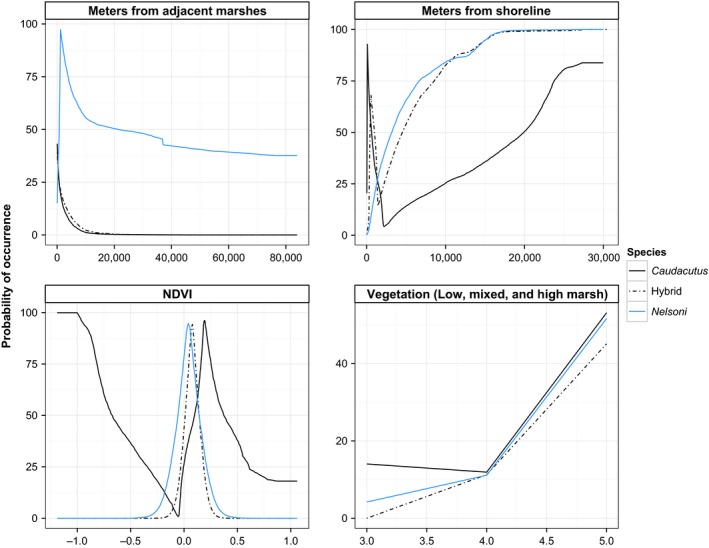
Occurrence probabilities for pure *caudacutus*, pure *nelsoni*, and hybrids for each of the four habitat variables used in the ecological niche models.

Ecological niche models for pure species and hybrids performed better than random, resulting in mean AUC values >0.7 (*A. nelsoni* – mean ± SD = 0.800 ± 0.055; *A. caudacutus* = 0.741 ± 0.089; hybrids = 0.792 ± 0.127). The relative contribution of vegetation composition, marsh isolation, and shoreline distance to the niche models varied for the three groups (Table 5). Shoreline distance made a strong relative contribution to the Maxent models for both pure *caudacutus* and *nelsoni* (47.9% and 35.6%, respectively; Table 5). The relative contribution of vegetation composition (high marsh, mixed marsh, and low marsh; 35.6%; *nelsoni*, 19%; *caudacutus*) and proximity index (17.3%; *nelsoni*, 30.9%; *caudacutus*) varied across the pure niche models. NDVI was most important for the hybrid niche models (44.3%), followed by vegetation composition (29.9%) and distance to shoreline (13.3%). The probability of occurrence, based on individual habitat variables, fluctuated for each group with hybrids intermediate between the two pure taxa (Fig. 7). Habitat suitability varied between pure taxa: *nelsoni* had a higher probability of occurring in dry marshes, farther from the ocean, while *caudacutus* occurred in both wet and dry marshes that were larger, more connected, and closer to the coast. In some instances, hybrids showed similarities to pure *caudacutus* and in other instances, the hybrid occurrence probabilities mirrored patterns more closely found in pure *nelsoni*. Hybrids were similar to *nelsoni* with a higher probability of occurrence in dry marshes farther from the ocean and similar to *caudacutus* with a higher probability of occurrence in more connected marshes.

**Table 5:** Contribution of environmental variables to the *nelsoni*,* caudacutus*, and hybrid ecological niche models

Variable	% Contribution to niche models		
	*A. nelsoni*	*A. caudacutus*	Hybrids
NDVI	14	2.1	44.3
Vegetation	35.6	19	29.9
Shoreline distance	33.1	47.9	13.3
Proximity index	17.3	30.9	12.5

**Figure 8:**
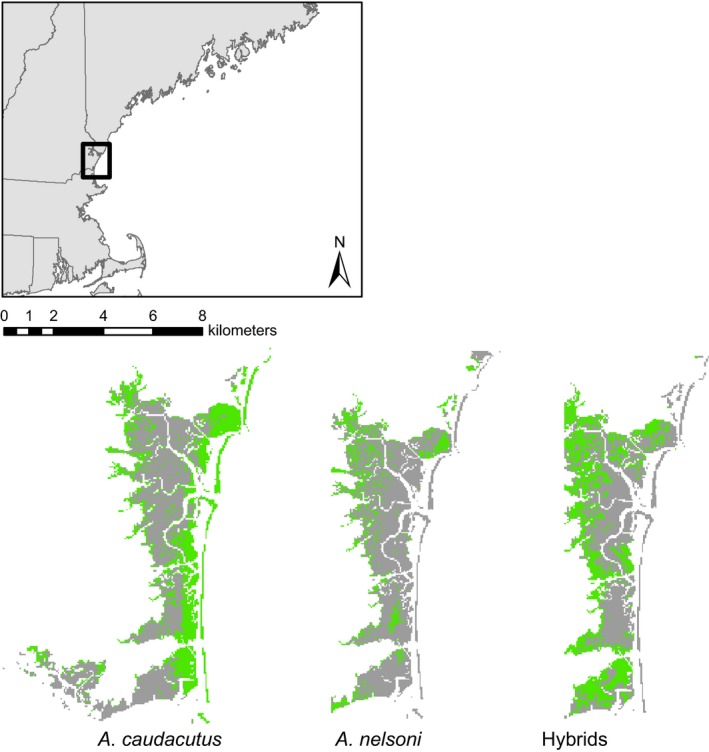
Representative example of suitable habitat predictions for pure *caudacutus,* pure *nelsoni*, and hybrids in one marsh complex (Hampton/Salisbury marsh in New Hampshire). Suitable habitat is shown in green.

**Figure 9:**
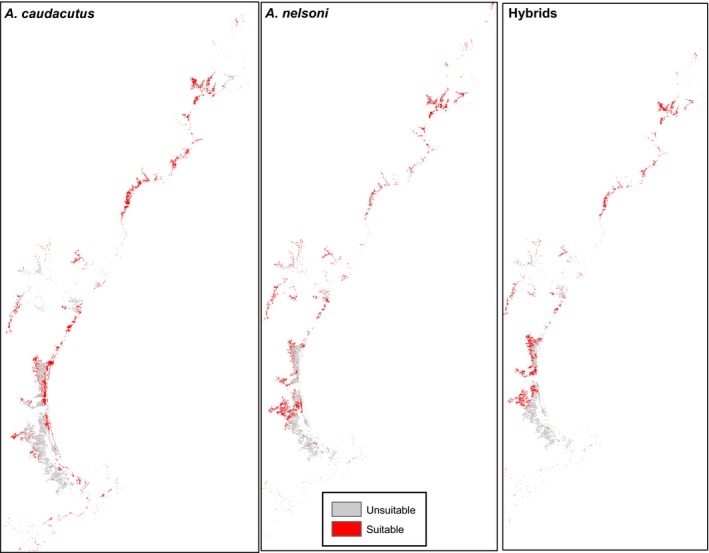
Maxent output for a portion of the study area. Cumulative output was averaged from 10 Maxent runs and split into suitable (red) and unsuitable (gray) habitat using a threshold value (lower 5th percentile of the distribution of cumulative probabilities for each group) cumulative probabilities for each group: pure caudacutus, nelsoni, and hybrids.

When comparing known occurrences to the predicted distributions (categorized as suitable versus unsuitable based on cumulative thresholds), the niche models matched the observed data well. A high percentage of the known occurrence points (78% of *nelsoni*, 82% of *caudacutus*, and 80% of hybrid points) fell within habitat predicted to be suitable by Maxent. There were observable differences in the distribution maps for *nelsoni* and *caudacutus,* with *nelsoni* more commonly predicted up river and *caudacutus* predicted along the coast (Figs. 8, 9). Substantial niche overlap was evident among the three groups. While *nelsoni* and *caudacutus* occupied similar niches, there were some differences in niche type occupied by the two parental groups (Schoener's *D* = 0.78; Warren's *I* = 0.95). Hybrids showed greater niche overlap with *nelsoni* (*D* = 0.88; *W* = 0.989) than with *caudacutus* (*D* = 0.81; *W* = 0.96). Similar to the pure *nelsoni* group, hybrids appeared to be more commonly predicted farther away from the coastline (Fig. 8).

## Discussion

Our study offers support for local habitat features in shaping hybrid zone dynamics across a tidal marsh gradient and suggests a potential role for habitat divergence and salt marsh adaptation as an isolating mechanism between two avian sister species. As predicted, the complex spatial structuring of parental taxa and hybrids observed in this system offers support for a mosaic hybrid zone. Our findings contribute to a growing body of literature supporting the importance of local habitat features and environment‐dependent selection in shaping natural hybrid zones (Carson et al. 2012; Tarroso et al. 2014). Recent studies have found increasing evidence for spatial structuring across numerous ecological gradients, including temperature gradients (Culumber et al. 2012), vegetation/substrate gradients (Shurtliff et al. 2013), bioclimatic gradients (Tarroso 2014), and elevational gradients (DuBay and Witt 2014). This study offers empirical evidence for exogenous selection in a novel ecotone, providing support for hybrid zone maintenance along tidal marsh gradients. Our findings do not preclude an additional role for endogenous factors, such as reduction in hybrid fitness, and indeed multiple forces may be acting simultaneously in this system (Walsh 2015).

Environmental variation explained the spatial distribution of *A. caudacutus* and *A. nelsoni* genotypes across our sampling area. There was a positive correlation between site‐averaged genotype and habitat variables, which appears to be largely driven by tidal regime (as predicted by vegetation) within a marsh patch and more general marsh features (size, isolation, distance to shoreline) at the marsh‐complex scale. Comparison of suitability predictions from ecological niche models further shows that *caudacutus*,* nelsoni*, and their hybrids display slight differences in niche breadth, despite broad similarity in habitat suitability. We detected marked differences in habitat type between allopatric *nelsoni* and allopatric *caudacutus* populations. Pure *nelsoni* marshes were generally characterized as small, isolated, brackish river marshes in comparison to pure *caudacutus* marshes, which were larger, more connected, and saline coastal marshes. These findings are consistent with previous observations of habitat differences between *A. caudacutus* and *A. nelsoni* (Greenlaw 1993; Greenlaw and Rising 1994). While the hybrid zone cannot be characterized as an intermediate habitat type (or ecotone) in the more traditional sense, it does display a higher diversity of marsh types than found to the north and south of the zone (based on the distribution of habitat PC scores). The diversity of habitat within the hybrid zone thus likely facilitates the co‐habitation of pure individuals and creates increased opportunities for hybridization.

The extent to which local environmental features influence introgression is dependent on how restricted a species is to a habitat and how that habitat is distributed across the landscape (Nosil et al. 2005; Shurtliff et al. 2013). Our results suggest that local marsh characteristics shape the distribution of *nelsoni* and *caudacutus* individuals, and their hybrids, either due to active habitat preferences or differential adaptation. These findings are supported by genomic cline analyses showing differential selection for traits related to salt marsh adaptations (Walsh et al. in review). We argue that the observed distributions cannot be explained by geographic location alone, as marshes differ in genotypic composition even over short distances. For example, at Popham Beach, Maine (sampling location 8) we identified a mix of genotypes (pure individuals and both backcrossed and recent generation hybrids), while approximately 20 km away Maquoit Bay, Maine (sampling location 9) was comprised of only pure and backcrossed *nelsoni*. Based on habitat data (point‐of‐capture scale), both Popham Beach and Maquoit Bay were dry with an abundance of high marsh (100% and 62%, respectively) in areas where the birds were sampled. This translates into nesting habitat that is suitable for both *caudacutus* and *nelsoni*. One key difference between Popham Beach and Maquoit Bay, however, is the difference in size and degree of isolation. Popham Beach is larger and more connected (143 ha, proximity index of 0.25) compared to Maquoit Bay (28 ha, proximity index of 0.034). Maquoit Bay is also more sheltered and less tidally influenced compared to Popham Beach, which is a coastal marsh. While 28 ha is not too small to support *caudacutus* populations (Benoit and Askins 2002), the vegetation at Popham Beach combined with the size and connectivity of the marsh may provide suitable habitat for both species, whereas the vegetation is suitable at Maquoit Bay but the marsh is too isolated for *caudacutus,* thus explaining the observed patterns.

Further support for a mosaic pattern across the *caudacutus‐nelsoni* hybrid zone comes from the observed distribution of genotypic classes (pure, F1/F2, and backcrossed individuals) within the major marsh types (coastal, river, and intermediate classifications based on habitat PC1 scores). We found that coastal marshes are comprised predominantly of pure and backcrossed *caudacutus* individuals (94% of individuals in coastal sites were from these two genotypic classes). This is in contrast with genotypic composition within the intermediate and river marshes, where we observed predominantly *nelsoni* individuals (pure and backcrossed), F1/F2 hybrids, and backcrossed *caudacutus*. The proportion of pure *caudacutus* was relatively low in intermediate and river marshes, comprising 13% and 17% of individuals in these sites, respectively. Based on our findings, it seems likely that rates of hybridization and introgression vary among marsh patches based on local habitat characteristics.

Arguably, limits to *nelsoni* reproductive success in coastal marshes may contribute to some degree of habitat isolation (Nosil 2012). Although the drivers of habitat selection are less clear in *caudacutus*, we did detect comparatively fewer pure *caudacutus* individuals in river and intermediate marshes compared to coastal marshes. Furthermore, while we found *caudacutus* individuals in intermediate and river sites, a high percentage of the birds were backcrossed as opposed to pure (comprising 36% of individuals in intermediate marshes and 25% in river marshes). It is possible that while the habitat may be suitable for nesting in the upriver sites, the isolation of some of the river marshes within our study area makes them less accessible to pure *caudacutus*, if they follow a coastal migration pattern, which may be expected for birds breeding in tidal marshes. Despite the isolation of river marshes, the presence of backcrossed *caudacutus* likely prevents complete reproductive isolation and may lead to increased admixture in river and intermediate habitats.

The spatial distribution of tidal marshes within our study area may further play a role in shaping hybrid zone boundaries and future trajectories of hybridization between pure *caudacutus* and *nelsoni*. Mosaic hybrid zones may facilitate rapid genetic swamping in cases where pockets of the rare species are found within a matrix of a more common species (Dabrowski et al. 2005). Alternatively, strong habitat preferences, and differential fitness across habitat types, may provide refugia for pure individuals (Confer et al. 2010; Aldinger and Wood 2014) and limit the frequency of hybridization events. Based on our findings, we predict that the local marsh features will limit the extent to which pure *nelsoni* and *caudacutus* individuals overlap. However, backcrossing appears to be frequent and the introgression of parental alleles is not limited or bounded by the transition between marsh types along the coastline. Therefore, while environmental forces may limit the distribution of pure species, introgression may continue well beyond the limits of the hybrid zone, as dispersal and backcrossing facilitate interspecific gene flow.

## Conflict of Interest

Authors have no conflict of interest to declare.

## Supporting information


**Appendix S1.** Interspecific heterozygosity plotted against hybrid index for 237 individuals sampled from putatively sympatric populations.
**Appendix S2.** Geographic coordinates for occurrence points used in ecological niche models.
