# Supplementary material for: Genotype‐environment associations support a mosaic hybrid zone between two tidal marsh birds
Source: Ecol Evol. 2015 Dec 29;6(1):279–94. doi: 10.1002/ece3.1864 (PMC4716509; doi:10.1002/ece3.1864)
Supplement: Supplementary file 1 — Appendix S1. Interspecific heterozygosity plotted against hybrid index for 237 individuals sampled from putatively sympatric populations. Appendix S2. Geographic coordinates for occurrence points used in ecological niche models. [file ECE3-6-279-s001.docx]

**Appendix S1**

Interspecific heterozygosity plotted against hybrid index for 237 individuals sampled from putatively sympatric populations. Colors represent assigned genotypic classes, which were determined by comparing hybrid index and interspecific heterozygosity for each individual. Individuals with intermediate hybrid index (0.25-0.75) and high heterozygosity (>0.3) were considered recent generation hybrids and individuals with low hybrid index (<0.25 or >0.75) and low heterozygosity (<0.3) were considered backcrossed.

**Appendix S2**

Geographic coordinates for occurrence points used in ecological niche models. All points are organized by group – pure *caudacutus*, *pure nelsoni*, and admixed.

| Individual | Population | X (UTM) | Y (UTM) |
| --- | --- | --- | --- |
| S1 | *Ammodramus caudacutus* | 386997.17 | 4813452.01 |
| S2 | *Ammodramus caudacutus* | 346621.03 | 4768589.95 |
| S3 | *Ammodramus caudacutus* | 355470.88 | 4722214.32 |
| S4 | *Ammodramus caudacutus* | 333919.34 | 4685099.92 |
| S5 | *Ammodramus caudacutus* | 333559.89 | 4684080.95 |
| S6 | *Ammodramus caudacutus* | 359573.02 | 4653120.00 |
| S7 | *Ammodramus caudacutus* | 358730.40 | 4652169.81 |
| S8 | *Ammodramus caudacutus* | 358036.45 | 4651852.55 |
| S9 | *Ammodramus caudacutus* | 359748.06 | 4652723.65 |
| S10 | *Ammodramus caudacutus* | 363184.18 | 4616700.57 |
| S11 | *Ammodramus caudacutus* | 297397.03 | 4618272.94 |
| S12 | *Ammodramus caudacutus* | 303288.94 | 4620909.59 |
| S13 | *Ammodramus caudacutus* | 298771.02 | 4618174.99 |
| S14 | *Ammodramus caudacutus* | 313906.08 | 4619268.99 |
| S15 | *Ammodramus caudacutus* | 308902.03 | 4616125.95 |
| S16 | *Ammodramus caudacutus* | 308808.49 | 4616916.56 |
| S17 | *Ammodramus caudacutus* | 309125.83 | 4616631.21 |
| S18 | *Ammodramus caudacutus* | 306451.87 | 4610749.66 |
| S19 | *Ammodramus caudacutus* | 311759.76 | 4611223.78 |
| S20 | *Ammodramus caudacutus* | 310453.66 | 4612167.35 |
| S21 | *Ammodramus caudacutus* | 309726.69 | 4612554.91 |
| S22 | *Ammodramus caudacutus* | 309608.14 | 4613103.50 |
| S23 | *Ammodramus caudacutus* | 306691.14 | 4611344.11 |
| S24 | *Ammodramus caudacutus* | 315705.88 | 4601780.14 |
| S25 | *Ammodramus caudacutus* | 316186.91 | 4601558.86 |
| S26 | *Ammodramus caudacutus* | 376896.46 | 4579636.46 |
| S27 | *Ammodramus caudacutus* | 377352.09 | 4582367.76 |
| S28 | *Ammodramus caudacutus* | 378217.56 | 4581082.05 |
| S29 | *Ammodramus caudacutus* | 374576.18 | 4581650.00 |
| S30 | *Ammodramus caudacutus* | 373262.88 | 4584360.52 |
| S31 | *Ammodramus caudacutus* | 374433.15 | 4581225.58 |
| S32 | *Ammodramus caudacutus* | 373387.19 | 4583823.27 |
| S33 | *Ammodramus caudacutus* | 350666.67 | 4578519.93 |
| S34 | *Ammodramus caudacutus* | 389129.18 | 4811470.63 |
| S35 | *Ammodramus caudacutus* | 354477.72 | 4726589.13 |
| S36 | *Ammodramus caudacutus* | 381088.03 | 4622163.95 |
| S37 | *Ammodramus caudacutus* | 382063.80 | 4620676.62 |
| S38 | *Ammodramus caudacutus* | 381777.18 | 4620972.54 |
| S39 | *Ammodramus caudacutus* | 382992.43 | 4620871.94 |
| S40 | *Ammodramus caudacutus* | 383843.61 | 4620434.51 |
| S41 | *Ammodramus caudacutus* | 388712.94 | 4617730.73 |
| S42 | *Ammodramus caudacutus* | 388264.17 | 4617841.55 |
| S43 | *Ammodramus caudacutus* | 389037.04 | 4618135.49 |
| S44 | *Ammodramus caudacutus* | 388303.38 | 4610678.02 |
| S45 | *Ammodramus caudacutus* | 390137.68 | 4609464.02 |
| S46 | *Ammodramus caudacutus* | 389812.17 | 4610192.29 |
| S47 | *Ammodramus caudacutus* | 307068.67 | 4627176.74 |
| S48 | *Ammodramus caudacutus* | 309848.54 | 4626068.97 |
| S49 | *Ammodramus caudacutus* | 308237.29 | 4626483.81 |
| S50 | *Ammodramus caudacutus* | 307333.06 | 4626718.92 |
| S51 | *Ammodramus caudacutus* | 309702.55 | 4625556.96 |
| S52 | *Ammodramus caudacutus* | 306976.03 | 4627577.92 |
| S53 | *Ammodramus caudacutus* | 310349.86 | 4625251.31 |
| S54 | *Ammodramus caudacutus* | 309830.12 | 4623264.75 |
| S55 | *Ammodramus caudacutus* | 309628.65 | 4620424.08 |
| S56 | *Ammodramus caudacutus* | 308712.37 | 4620297.39 |
| S57 | *Ammodramus caudacutus* | 308432.13 | 4620014.54 |
| S58 | *Ammodramus caudacutus* | 305192.08 | 4623141.99 |
| S59 | *Ammodramus caudacutus* | 373960.97 | 4601187.95 |
| S60 | *Ammodramus caudacutus* | 316371.62 | 4605343.08 |
| S61 | *Ammodramus caudacutus* | 315036.25 | 4603356.65 |
| S62 | *Ammodramus caudacutus* | 316600.05 | 4606079.98 |
| S63 | *Ammodramus caudacutus* | 315570.01 | 4602943.98 |
| S64 | *Ammodramus caudacutus* | 371544.75 | 4584813.37 |
| S65 | *Ammodramus caudacutus* | 369173.26 | 4587155.20 |
| S66 | *Ammodramus caudacutus* | 369384.67 | 4588211.11 |
| S67 | *Ammodramus caudacutus* | 488630.01 | 4880434.92 |
| S68 | *Ammodramus caudacutus* | 452188.31 | 4879265.53 |
| S69 | *Ammodramus caudacutus* | 435096.97 | 4843200.44 |
| S70 | *Ammodramus caudacutus* | 407015.92 | 4851445.43 |
| S71 | *Ammodramus caudacutus* | 399066.18 | 4826512.34 |
| S72 | *Ammodramus caudacutus* | 390146.15 | 4824516.21 |
| S73 | *Ammodramus caudacutus* | 387530.09 | 4816392.65 |
| S74 | *Ammodramus caudacutus* | 383922.10 | 4804122.74 |
| S75 | *Ammodramus caudacutus* | 375252.45 | 4800167.29 |
| S76 | *Ammodramus caudacutus* | 372476.31 | 4794441.48 |
| S77 | *Ammodramus caudacutus* | 345071.40 | 4770678.80 |
| S78 | *Ammodramus caudacutus* | 343280.18 | 4767164.09 |
| S79 | *Ammodramus caudacutus* | 342310.92 | 4764715.80 |
| S80 | *Ammodramus caudacutus* | 357209.68 | 4762970.39 |
| S81 | *Ammodramus caudacutus* | 352617.58 | 4754179.50 |
| S82 | *Ammodramus caudacutus* | 351114.70 | 4745101.54 |
| S83 | *Ammodramus caudacutus* | 352010.19 | 4737305.21 |
| N1 | *Ammodramus nelsoni* | 641732.09 | 4971983.48 |
| N2 | *Ammodramus nelsoni* | 643514.07 | 4971189.47 |
| N3 | *Ammodramus nelsoni* | 645321.57 | 4971315.76 |
| N4 | *Ammodramus nelsoni* | 651502.51 | 4968265.71 |
| N5 | *Ammodramus nelsoni* | 651267.52 | 4967823.37 |
| N6 | *Ammodramus nelsoni* | 651313.74 | 4967217.82 |
| N7 | *Ammodramus nelsoni* | 651008.26 | 4968552.49 |
| N8 | *Ammodramus nelsoni* | 652587.63 | 4959136.38 |
| N9 | *Ammodramus nelsoni* | 627800.58 | 4953282.58 |
| N10 | *Ammodramus nelsoni* | 625783.06 | 4952941.97 |
| N11 | *Ammodramus nelsoni* | 621904.72 | 4953964.59 |
| N12 | *Ammodramus nelsoni* | 626092.66 | 4952659.99 |
| N13 | *Ammodramus nelsoni* | 622591.01 | 4953616.46 |
| N14 | *Ammodramus nelsoni* | 624661.95 | 4952102.33 |
| N15 | *Ammodramus nelsoni* | 624451.94 | 4952629.22 |
| N16 | *Ammodramus nelsoni* | 623467.21 | 4952605.40 |
| N17 | *Ammodramus nelsoni* | 623983.13 | 4952826.32 |
| N18 | *Ammodramus nelsoni* | 621708.38 | 4942032.38 |
| N19 | *Ammodramus nelsoni* | 621593.76 | 4941577.30 |
| N20 | *Ammodramus nelsoni* | 621691.87 | 4942787.46 |
| N21 | *Ammodramus nelsoni* | 621071.60 | 4943324.75 |
| N22 | *Ammodramus nelsoni* | 609076.17 | 4932618.63 |
| N23 | *Ammodramus nelsoni* | 510726.13 | 4937808.56 |
| N24 | *Ammodramus nelsoni* | 510830.58 | 4937055.39 |
| N25 | *Ammodramus nelsoni* | 578832.68 | 4920467.53 |
| N26 | *Ammodramus nelsoni* | 578858.40 | 4919960.59 |
| N27 | *Ammodramus nelsoni* | 579050.15 | 4919477.55 |
| N28 | *Ammodramus nelsoni* | 578409.56 | 4921351.05 |
| N29 | *Ammodramus nelsoni* | 578367.64 | 4920906.07 |
| N30 | *Ammodramus nelsoni* | 511066.02 | 4933753.74 |
| N31 | *Ammodramus nelsoni* | 574444.26 | 4913501.73 |
| N32 | *Ammodramus nelsoni* | 489303.11 | 4881043.34 |
| N33 | *Ammodramus nelsoni* | 489004.80 | 4880663.09 |
| N34 | *Ammodramus nelsoni* | 488864.88 | 4879642.59 |
| N35 | *Ammodramus nelsoni* | 468404.19 | 4879154.61 |
| N36 | *Ammodramus nelsoni* | 488955.08 | 4879039.34 |
| N37 | *Ammodramus nelsoni* | 489349.97 | 4879027.58 |
| N38 | *Ammodramus nelsoni* | 453352.44 | 4875128.67 |
| N39 | *Ammodramus nelsoni* | 452743.78 | 4875063.02 |
| N40 | *Ammodramus nelsoni* | 453070.06 | 4874738.24 |
| N41 | *Ammodramus nelsoni* | 451285.49 | 4874646.08 |
| N42 | *Ammodramus nelsoni* | 452241.52 | 4873719.30 |
| N43 | *Ammodramus nelsoni* | 451302.54 | 4873653.06 |
| N44 | *Ammodramus nelsoni* | 448826.08 | 4872512.60 |
| N45 | *Ammodramus nelsoni* | 397300.31 | 4825588.91 |
| N46 | *Ammodramus nelsoni* | 397101.63 | 4824261.02 |
| N47 | *Ammodramus nelsoni* | 387690.35 | 4813402.53 |
| N48 | *Ammodramus nelsoni* | 386062.56 | 4814439.02 |
| N49 | *Ammodramus nelsoni* | 386775.37 | 4817135.51 |
| N50 | *Ammodramus nelsoni* | 601194.54 | 4941470.00 |
| N51 | *Ammodramus nelsoni* | 600608.75 | 4941285.65 |
| N52 | *Ammodramus nelsoni* | 601552.39 | 4942074.15 |
| N53 | *Ammodramus nelsoni* | 601419.68 | 4943467.91 |
| N54 | *Ammodramus nelsoni* | 601760.63 | 4943897.15 |
| N55 | *Ammodramus nelsoni* | 601567.85 | 4944383.44 |
| N56 | *Ammodramus nelsoni* | 601623.29 | 4943086.23 |
| N57 | *Ammodramus nelsoni* | 586218.41 | 4936128.77 |
| N58 | *Ammodramus nelsoni* | 586460.17 | 4935614.27 |
| N59 | *Ammodramus nelsoni* | 586803.29 | 4935019.03 |
| N60 | *Ammodramus nelsoni* | 590671.77 | 4933662.67 |
| N61 | *Ammodramus nelsoni* | 591105.92 | 4933465.99 |
| N62 | *Ammodramus nelsoni* | 587192.15 | 4934648.07 |
| N63 | *Ammodramus nelsoni* | 590209.17 | 4933622.44 |
| N64 | *Ammodramus nelsoni* | 587698.53 | 4934160.06 |
| N65 | *Ammodramus nelsoni* | 588293.63 | 4933509.31 |
| N66 | *Ammodramus nelsoni* | 511120.01 | 4936494.27 |
| N67 | *Ammodramus nelsoni* | 511259.24 | 4936133.06 |
| N68 | *Ammodramus nelsoni* | 511330.25 | 4936843.31 |
| N69 | *Ammodramus nelsoni* | 522533.25 | 4923889.29 |
| N70 | *Ammodramus nelsoni* | 521243.72 | 4923690.56 |
| N71 | *Ammodramus nelsoni* | 517724.16 | 4910733.38 |
| N72 | *Ammodramus nelsoni* | 488532.15 | 4879504.33 |
| N73 | *Ammodramus nelsoni* | 488555.29 | 4879043.46 |
| N74 | *Ammodramus nelsoni* | 482329.40 | 4881541.17 |
| N75 | *Ammodramus nelsoni* | 482639.09 | 4880214.06 |
| N76 | *Ammodramus nelsoni* | 434779.02 | 4842928.63 |
| N77 | *Ammodramus nelsoni* | 390890.94 | 4822010.20 |
| N78 | *Ammodramus nelsoni* | 396458.01 | 4824534.97 |
| N79 | *Ammodramus nelsoni* | 389711.00 | 4810671.91 |
| N80 | *Ammodramus nelsoni* | 488630.01 | 4880434.92 |
| N81 | *Ammodramus nelsoni* | 452188.31 | 4879265.53 |
| N82 | *Ammodramus nelsoni* | 435096.97 | 4843200.44 |
| N83 | *Ammodramus nelsoni* | 420611.08 | 4857575.36 |
| N84 | *Ammodramus nelsoni* | 407712.74 | 4853272.55 |
| N85 | *Ammodramus nelsoni* | 399066.18 | 4826512.34 |
| N86 | *Ammodramus nelsoni* | 390146.15 | 4824516.21 |
| N87 | *Ammodramus nelsoni* | 387530.09 | 4816392.65 |
| N88 | *Ammodramus nelsoni* | 383922.10 | 4804122.74 |
| N89 | *Ammodramus nelsoni* | 375252.45 | 4800167.29 |
| N90 | *Ammodramus nelsoni* | 372476.31 | 4794441.48 |
| N91 | *Ammodramus nelsoni* | 345071.40 | 4770678.80 |
| N92 | *Ammodramus nelsoni* | 343280.18 | 4767164.09 |
| N93 | *Ammodramus nelsoni* | 342310.92 | 4764715.80 |
| N94 | *Ammodramus nelsoni* | 357209.68 | 4762970.39 |
| A1 | Admixed | 452189 | 4879331 |
| A2 | Admixed | 435291 | 4843309 |
| A3 | Admixed | 420533 | 4857628 |
| A4 | Admixed | 375310 | 4800147 |
| A5 | Admixed | 344993 | 4770721 |
| A6 | Admixed | 488513 | 4880519 |
| A7 | Admixed | 435999 | 4851800 |
| A8 | Admixed | 390237 | 4824587 |
| A9 | Admixed | 406912 | 4851704 |
| A10 | Admixed | 399182 | 4826964 |
| A11 | Admixed | 387514 | 4816629 |
| A12 | Admixed | 383883 | 4804260 |
| A13 | Admixed | 372460 | 4794490 |
| A14 | Admixed | 360131 | 4780470 |
| A15 | Admixed | 364580 | 4771880 |
| A16 | Admixed | 342431 | 4764701 |
| A17 | Admixed | 343266 | 4767227 |
| A18 | Admixed | 348682 | 4754797 |
| A19 | Admixed | 357024 | 4762875 |
| A20 | Admixed | 352442 | 4754372 |
| A21 | Admixed | 351244 | 4745248 |
| A22 | Admixed | 350299 | 4738231 |
| A23 | Admixed | 351996 | 4737490 |
